# Key Techniques and Risk Management for the Application of the Pile-Beam-Arch (PBA) Excavation Method: A Case Study of the Zhongjie Subway Station

**DOI:** 10.1155/2014/275362

**Published:** 2014-04-09

**Authors:** Yong-ping Guan, Wen Zhao, Shen-gang Li, Guo-bin Zhang

**Affiliations:** ^1^School of Resources and Civil Engineering, Northeastern University, Shenyang 110819, China; ^2^Shenyang Design and Research Institute of Municipal Engineering, Shenyang 110015, China

## Abstract

The design and construction of shallow-buried tunnels in densely populated urban areas involve many challenges. The ground movements induced by tunneling effects pose potential risks to infrastructure such as surface buildings, pipelines, and roads. In this paper, a case study of the Zhongjie subway station located in Shenyang, China, is examined to investigate the key construction techniques and the influence of the Pile-Beam-Arch (PBA) excavation method on the surrounding environment. This case study discusses the primary risk factors affecting the environmental safety and summarizes the corresponding risk mitigation measures and key techniques for subway station construction using the PBA excavation method in a densely populated urban area.

## 1. Introduction

Subway construction projects are generally located in complex surrounding environments and are often built beside or under residentially, commercially, or officially important buildings. However, tunneling in dense urban areas can cause ground movements and surface settlements, which may lead to additional deformations and damage to existing structures and utilities such as residential buildings and pipelines [1]. In this situation, one of the key factors in design and construction problems may be the amount of allowable settlement.

Underground projects are extremely complex and are associated with many uncertainties resulting from geological and geomechanical parameters, external load, and construction quality [3]. These uncertainties during tunneling can lead to potential risks to both the workers and the surrounding environment [4]. To minimize the adverse effects on the surrounding environment and perform appropriate risk mitigation measures in time, a risk management technique should be adopted throughout the underground construction project development [5].

Many studies have been conducted to investigate the risk assessment of tunneling projects in urban areas and the adverse impact of the PBA excavation method on ground settlement and adjacent utilities.

Considering the uncertainties, Einstein [6] proposed a probabilistic approach to assist decision making in the form of engineering design, selecting particular construction procedures or more general decisions made by decision makers in geotechnical engineering. Reilly [7] discussed an overview of management for complex, underground, and tunneling projects, suggested an improved methodology for the “project delivery process,” and summarized crucial supporting systems such as partnering and risk mitigation. You et al. [8] presented a methodology to select an optimal supporting scheme and advance rate quantitatively for the design of a tunnel by performing a risk analysis considering the construction fee and the cost of losses related to tunnel collapse. Fang et al. [9] proposed a risk management methodology that aims at process control for ground settlement and surface buildings, to guarantee the environmental safety.

In terms of the PBA excavation method research, Wang et al. [10–12] analyzed the influence of a subway station constructed by the PBA excavation method on the ground settlements and adjacent pipelines by means of numerical simulation and field measurements. Yang et al. [13] optimized the PBA construction procedure by implementing different schemes for heading opening patterns and heading excavation sequences based on three-dimensional numerical modelling. He [14] studied the main theoretical problems encountered during construction and the influence of metro tunneling using the PBA method on adjacent piles using the numerical simulation method.

However, there is limited research about the environmental risk assessment for subway station construction using the PBA (Pile-Beam-Arch) excavation method. This paper presents an in-depth investigation of the influence of the PBA excavation method on ground movements and the surrounding environment. Simultaneously, the potential risk encountered in this project during construction and corresponding risk mitigation measures are elaborated in detail. It is helpful to provide a valuable experience for other shallow-buried subway station construction projects in densely populated urban areas.

## 2. Project Overview

### 2.1. Geographic Location

**Figure 1:**
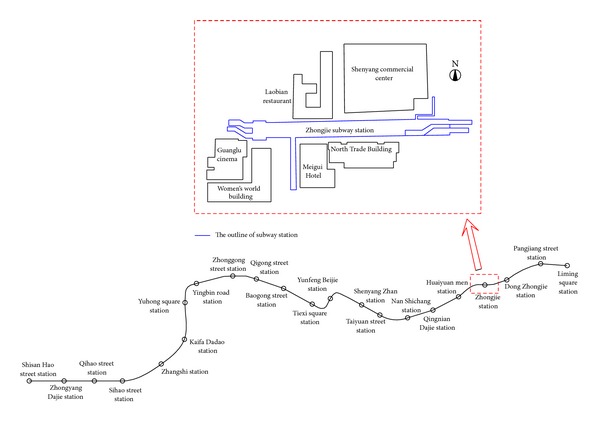
Main route of Shenyang Metro Line 1.

The first subway project constructed in Shenyang was the Metro Line 1 Project-Blue Line, China (see Figure 1). Metro Line 1 is almost 22 km in length with 22 subway stations. The line was located along jammed roads in the central city area as shown in Figure 1. The line runs from the Thirteen Street Station in the west to Li-Ming Square Station in the east. The strata in Shenyang along the tunnel alignment is comprised of numerous sandy layers with variable grain size distribution from silt sand to course gravel.

**Figure 2:**
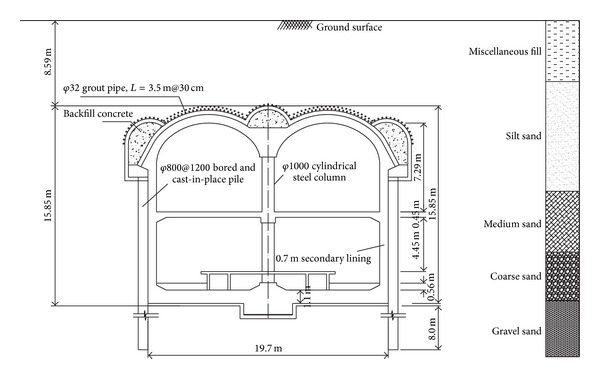
Typical cross section of Zhongjie subway station.

In this study, an environmental risk assessment was performed for the Zhongjie subway station, which was constructed using the PBA excavation method and is located in the chainage of DK17+880.567 to DK18+114.267. Zhongjie station is a double-deck tunnel of double-arch shape and the cross section size is approximately 19.7 m × 15.85 m. The overburden thickness is approximately 8.59 m, and the buried depth of the bottom is approximately 24.89 m. A typical cross section of the Zhongjie station is shown in Figure 2.

### 2.2. PBA Excavation Method

Urban subway stations are generally shallowly buried in China. Because of a thin overburden layer, a “soil arching effect” cannot be adequately formed above the tunnel roof [15]. Furthermore, the collapse surface of a shallow tunnel will easily extend from the tunnel face to the ground surface. However, the adjacent utilities (pipelines, crowded surface buildings) are significantly sensitive to ground movements induced by tunnel excavation, and excessive ground movements may induce damage to adjacent utilities. Plenty of successful subway construction projects in China have proved that the shallow tunneling method (STM) is very suitable for shallowly buried tunnel constructed in urban areas, which is inconvenient to excavate using the cut and cover method.

**Figure 3:**
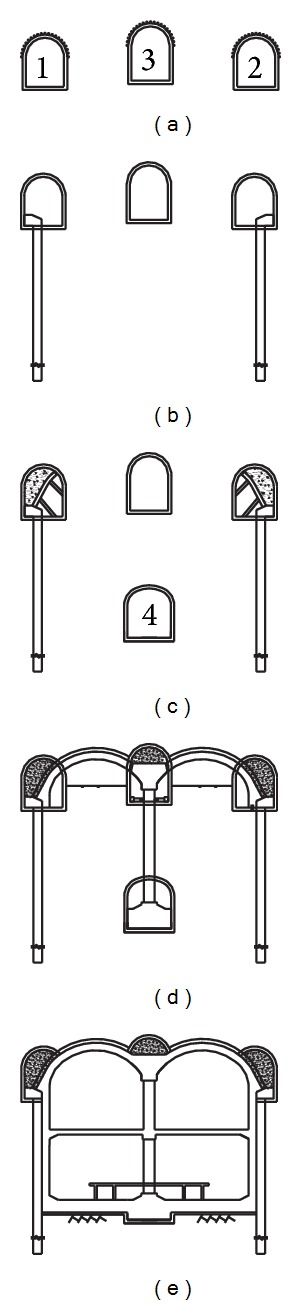
PBA excavation method adopted in the Zhongjie subway station.

The PBA (Pile-Beam-Arch) excavation method is one major approach to shallow tunneling and was adopted in the Zhongjie subway station construction project. Zhongjie subway station is a double-arch-double-span-double-deck station. The main steps of the PBA excavation method for the Zhongjie subway station are shown in Figure 3. The excavation sequence of the typical cross section can be divided into five steps. It is necessary to note that the PBA excavation process was implemented under dry conditions. Thus, an appropriate dewatering scheme should be designed to ensure that the groundwater table remains at least 0.5 m below the excavation bottom of the station during construction in similar situations.

**Figure 4:**
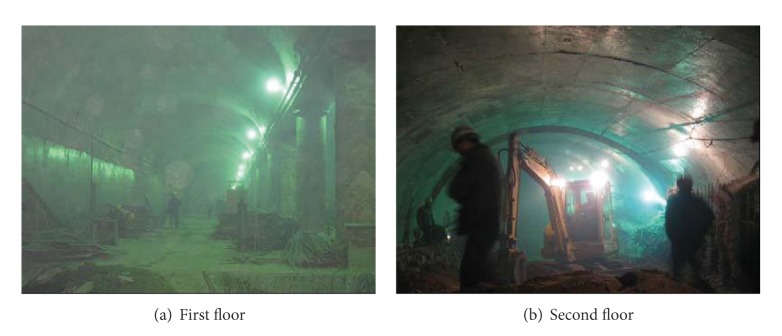
Photo of construction site.

The main construction sequences of the PBA excavation method are as follows:

1. upper pilot heading excavation using a multiface tunneling method (see Figure 3(a));
2. concreting the side piles (see Figure 3(b));
3. excavating the lower pilot heading (see Figure 3(c));
4. installing the middle steel column and constructing supporting arch (see Figure 3(d));
5. excavating the main structure via a top-down construction method and concreting secondary lining (see Figure 3(e)). The construction site situation is shown in Figure 4.

## 3. Numerical Investigation of the PBA Method

### 3.1. Three-Dimensional Modelling of Tunnel

The PBA excavation procedure was investigated with a three-dimensional simulation implemented using Flac3D code according to the finite difference method. The numerical simulation was capable of investigating the influence of PBA excavation processes on ground movements, which is helpful for providing a basis for the establishment of a process control index for ground surface settlements. In this study, the tunnel was assumed to be excavated in green field condition (ground surface above tunnel with no existence of buildings), and auxiliary methods such as sleeve-pipe grouting, forepoling pipe grouting, and feet-lock bolt were not considered. In addition, the following hypotheses were adopted in this study:

1. soil properties are assumed to be homogeneous and isotropic, with an elastic perfectly plastic constitutive relation with a Mohr-Coulomb yield criterion;
2. supporting structures such as lining and steel column are considered as elastic media.

The numerical simulation model was 50 m in the *z*-direction, 120 m (approximately 6D, D is the tunnel span) in the *x*-direction, and 60 m in the *y*-direction, and the number of grid cells was 741040 (as shown in Figure 6). The model was meshed in 50 longitudinal blocks of the same size. The tunnel linings were assumed to remain in contact with the surrounding soils and installed immediately after excavation. The hardening process of the sprayed concrete and the time delay effect of primary lining construction were considered by reducing the elastic modulus of the primary lining. In this study, the elastic modulus of the primary lining is 1/10 of that of its real value according to the suggestion given by Fang et al. [9].

As for boundary conditions, the horizontal displacements were set to zero at each side, which means that vertical displacements were allowed, and the node at the bottom of the mesh was fixed in both the vertical and horizontal directions. The top surface of the model was free in both directions.

**Figure 5:**
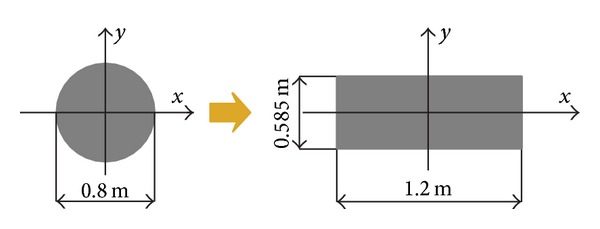
Modelling piles as a continuous wall.

**Figure 6:**
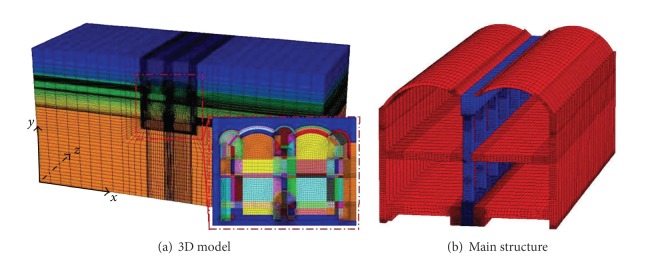
Finite element model.

In this project, the side pile's diameter is 0.8 m with a clearance of 0.4 m. By considering the importance of the bending rigidity of side pile, the equivalent values of EI were modelled in the rectangular cross section (see Figure 5). Thus, the equivalent thickness of the rectangular wall can be derived from (1) as follows:

(1) $$\begin{array}{c} \frac{1}{12}\left(D+t\right)h^{3}=\frac{1}{64}\pi D^{4}, \end{array}$$

where *D* is the pile's diameter, *t* is the pile spacing, which is 0.4 m in this project, and *h* is the equivalent thickness of the rectangular wall.

The soil properties and related parameters of the supporting structures used in this study are summarized in Table 1.

### 3.2. Results of the Numerical Simulation

For the subway station construction projects, especially in dense urban areas, it is necessary to prevent surface existing buildings and underground utilities from failing due to the ground movements induced by tunneling. The ground settlements should be strictly controlled, as excessive ground settlements may cause tunnel cave-in and cause negative effects or even damage to the existing surface buildings. Therefore, it is of paramount importance to investigate the influence area and degree as a response of a subway station construction using the PBA excavation method.

**Figure 7:**
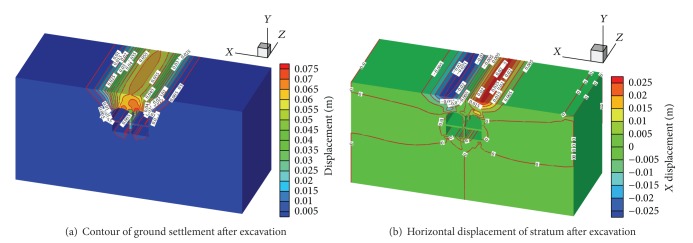
Displacement field obtained from the numerical simulation.

**Figure 8:**
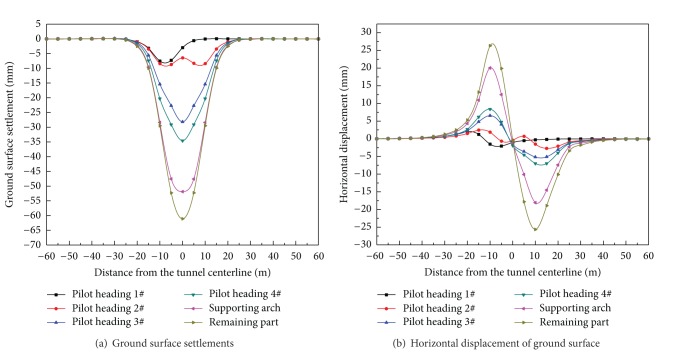
Ground movements induced by tunnel excavation in section *z* = −30 m.

The section at *z* = −30 m was chosen for investigation in this study. The strata settlement trough and ground movements are presented in Figures 7 and 8.  Figure 7 shows the simulated vertical displacement contour after tunnel excavation.  Figure 7(a) indicates that the width of the excavation influence region was found to be 3.0 times the tunnel span, approximately 60 m. This means the surface buildings located within a 30 m distance from the tunnel centerline would inevitably be affected by the tunneling activities and subject to differential settlements.

Figure 7(b) shows that the horizontal displacement of the ground surface was relatively symmetric about the tunnel centerline.

The influence of the PBA excavation method on the ground movements is shown in Figure 8, in which the progressive development of the transverse surface settlement trough and horizontal displacement of ground surface for the monitoring section *z* = −30 m are shown. The figure shows that the excavation influence zone extends symmetrically for approximately 30 m from the tunnel axis. As seen from Figure 8(a), the maximum ground settlement was approximately 61.1 mm after the excavation, appearing above the tunnel centerline. As shown in Figure 8(a), the additional ground surface settlement caused by the 1#, 2#, 3#, and 4# pilot heading excavation was 2.99 mm, 3.43 mm, 21.78 mm, and 6.4 mm, respectively. The maximum ground surface settlement was reached 34.6 mm after the excavation of pilot headings. In addition, the excavation of the supporting arch also has a great influence on the ground settlements, which caused 17.3 mm of additional settlement. The excavation of the four pilot headings and supporting arch had a major influence on the ground surface settlements, approximately 56.6% and 28.3% of additional settlement, respectively. This trend is similar to those reported by Wang et al. [11]. Such a trend indicates a requirement for closer attention to the stability of surface buildings when excavating pilot headings and supporting arches, especially during the period immediately after the installation of the sprayed concrete, which has not yet achieved its final stiffness.

As presented by Liu et al. [16], the horizontal displacement of ground surface induced by tunneling projects can also lead to the differential settlement and cracking of existing building. The horizontal displacement of ground surfaces caused by the PBA excavation method is shown in Figure 8(b), which is distributed in a wave shape. The horizontal displacements of ground surface were perfectly symmetrical about the tunnel centerline during the construction process. The maximum horizontal displacement was approximately 25 mm and appeared at 10 m distance from the tunnel centerline. The horizontal displacement of the ground surface induced by the pilot heading and supporting arch excavation constitutes a high proportion of the total displacement.

## 4. Potential Risks Identification

### 4.1. Groundwater Drawdown

The construction of an underground structure below the water table requires a strict and elaborate dewatering scheme. Groundwater may undermine construction safety since it induces additional loads on tunnel linings and decreases the soil strength [4]. The collapse of the Seoul subway tunnel [17] and that of a subway station in Shanghai have demonstrated the significant adverse effect of groundwater on underground projects. The presence of groundwater has increased the potential risks of underground projects.

There are rich groundwater resources in the Shenyang area, and the groundwater level is approximately 8 m below the ground surface. The Zhongjie station is located in sandy layers, which are strongly permeable, and the groundwater recharge velocity is fast. Thus, groundwater control is a key element in this project. The conventional handling of groundwater control may be performed using several methods: grouting, pumping, diaphragm wall, or a combination of them [18]. For subway construction projects, pumping wells have been extensively used in the Shenyang area because of the flexibility and low cost. However, the vast majority of pumping groundwater activities may lead to groundwater leakage and ground settlement due to the increase of soil effective stress. The design of the dewatering system should consider the environmental risks induced by groundwater drawdown.

### 4.2. Super-Shallow-Buried Depth

A general classification of tunnel cover-to-span is proposed by Wang [19] for the purpose of evaluation of the tunneling effect on ground settlement, based on the C/S ratio (cover-to-span). It is considered to be a shallow-buried tunnel when the C/S (cover-to-span) is within a range of 0.6–1.5, whereas, when the C/S is smaller than 0.6, it is considered to be a super-shallow-buried tunnel. For a deep-buried tunnel, there is a “soil arching effect” formed over the roof of the tunnel, which supports a large portion of overburden loads [20]. However, the mechanical behavior and deformation of the shallow-buried tunnel constructed in soft ground is significantly different from deep-buried tunnels, as the overburden layer is too thin to form a “soil arching effect” in shallow-buried tunnel [21].

In this project, the overburden layer is approximately 8.59 m, and the tunnel span is 19.7 m, for a C/S ratio of 0.43, a level at which it is difficult to produce a “soil arching effect.” Under this condition, the ground movements or collapse zones induced by excavation easily extend to the ground surface [22]. This unfavorable factor necessitated higher requirements for the tunnel excavation method, support patterns, water drainage, and grouting activities, and also increased the construction difficulty.

### 4.3. Existing Surface Buildings

**Figure 9:**
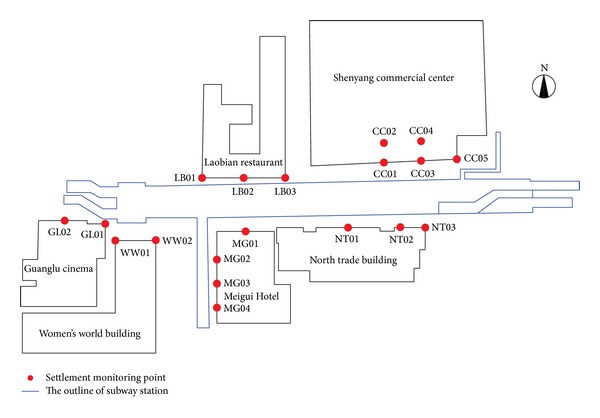
Zhongjie station and surface buildings relative locations.

Zhongjie subway station was constructed underneath a densely populated urban area, where many sensitive surface buildings might be affected by even minor variations in the foundation conditions, caused by either ground movements or dewatering activities. The location of surface buildings relative to the Zhongjie station is illustrated in Figure 9, which also shows the location of the settlement monitoring points for these surface buildings. The investigated detailed information about these surface buildings is summarized in Table 2.

These surface buildings were subjected to a detailed building condition investigation prior to the tunnel excavation, such as foundation type, age, height, and relative location to the subway station. According to the survey, these buildings are on different foundation types, that is, pile foundation, box foundation, and strip foundation, which were constructed in the last 15 years of the 20th century (see Table 2). Among all the cases, the Meigui Hotel is the oldest building and was constructed in 1987. The height of the building reaches 70 m, and it is adjacent to the temporary construction passage (#3 passageway) of the Zhongjie station. The distance between the exterior wall edge and excavation exterior edge is 2.67 m. The north side of the Meigui hotel is only 4.0 m away from the excavation exterior edge. Therefore, the Meigui Hotel is considered to be of the highest risk among surface buildings.

## 5. Environmental Risks Mitigation Measures

The utilization of isolation piles for ground movement control has been proved effective [23]. However, its disturbance and high construction cost have limited its application in practical engineering. For the Zhongjie station project, the implementation of isolation pile techniques is very difficult because of the limited space on the ground surface. Therefore, other risk mitigation measures, such as the sleeve-valve-pipe grouting technique, double-layer pipe grouting, and forepoling pipe grouting, are adopted to ensure the safety of tunnel construction and surrounding environment. All of these methods are implemented to minimize the ground movements induced by tunneling activities. More detailed information about these measures is elaborated as follows.

### 5.1. Groundwater Control

Dry tunneling conditions are one of the most important preconditions for PBA tunnel construction. The dewatering scheme should be designed to guarantee that the groundwater table remains at least 0.5 m below the excavation bottom during the excavation. In addition, the settlements of the ground surface and surface buildings caused by dewatering process should be controlled within an allowable range to mitigate the environmental risk during dewatering.

**Figure 10:**
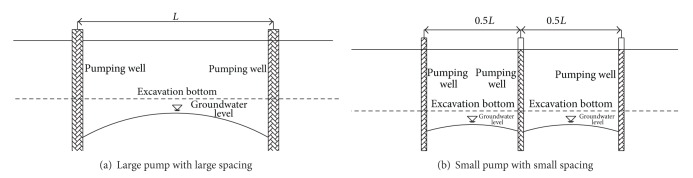
Details of pumping well layout.

In conventional dewatering approaches, the pumping well is always arranged using large spacing, a high pumping rate, and deep depth in the Shenyang area. As shown in Figure 10(a), the application of this dewatering scheme in subway station construction in densely populated urban areas has encountered some challenges.

1. To ensure the water table that between adjacent pumping wells remains at least 0.5 m below the excavation bottom during the excavation, the depths of the pumping well needed to be designed to be deep enough. This means a massive quantity of groundwater needs to be pumped, which leads to ground settlement and a substantial waste of expensive electricity.
2. Compared with normal building foundations, the subway station was composed of different building units, which had a different buried depth and excavation sequence. Thus, the application of a traditional dewatering scheme will pose potential difficulties for final-period management.
3. The subway station construction project has a characteristic of long construction period. Therefore, the long-term dewatering process requires high reliability and performance for these pumping wells. The groundwater level between adjacent pumping wells increases immediately once any one of these pumps breaks down because every single pumping well plays a good role in groundwater control.

Considering the above-mentioned shortcomings that exist in traditional dewatering scheme, in this project, the pumping well was arranged with small spacing (see Figure 10(b)). Compared to the above-mentioned dewatering scheme, it has the following advantages:

1. Under the same dewatering depth, the total amount of pumping water was decreased significantly, and the dewatering cost and environmental risks were also minimized.
2. Because of the small spacing, pumping wells can be flexibly arranged for different building units and construction sections. It is convenient to cease the dewatering activities for the sections where construction has been completed, and this is also helpful for decreasing the cost of dewatering.
3. Because more pumping wells are arranged around the station, the failure of a single pumping well has a limited impact on the whole dewatering system, with more time being allocated to maintaining the pumping well.

### 5.2. Pilot Heading Excavation Using a Multifaced Tunneling Method

**Figure 11:**
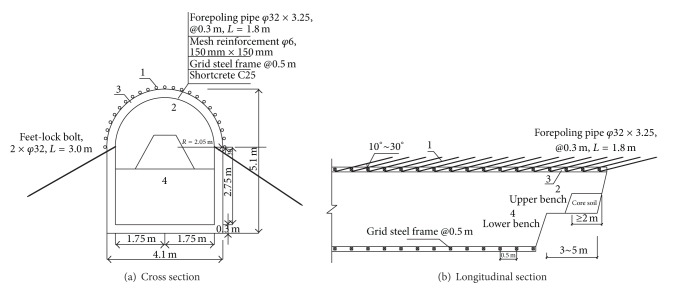
Excavation sequence of the pilot heading.

**Figure 12:**
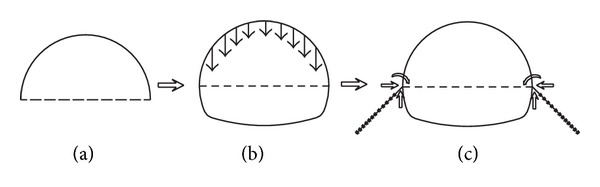
Mechanism of the feet-lock bolt (after [2]).

As mentioned in Section 3.2, the excavation of pilot headings had a major influence on the ground surface settlements. To restrict the ground movements induced by pilot heading excavation, some risk mitigation measures were implemented during pilot heading excavation. The forepoling pipe was installed prior to the excavation to improve the stability of the excavation face and the soil properties in the front of the tunnel face. The related parameters of the forepoling pipe used in this project are presented in Figure 11: *φ*32 mm with a thickness of 3.25 mm and a length of 1.8 m. These forepoling pipes were inserted at an angle of 10°–30° with the tunnel longitudinal direction into the soil above the arch ahead of the excavation face. The pilot heading was excavated using a multifaced tunneling method with short advance length. After the excavation of the upper bench, the overburden pressure exerts on the steel rib, which may lead to an integral sinking without a feet-lock bolt. Hence, the feet-lock bolts were installed immediately after excavating the upper bench to restrict the settlement and horizontal convergence of the steel rib. Luo and Chen [2] studied the complete set of the observation data collected from the tunnel construction project, and he reported that the feet-lock bolt was significantly effective in restricting the ground movement and grid steel rib's deformation in soft ground. He also illustrated the mechanism of the feet-lock bolt, as shown in Figure 12. The feet-lock bolt was subject to external forces such as bending moment, shear force, and axial force, which were transferred from the steel rib.

### 5.3. Sleeve-Valve-Pipe Grouting Technique

The sleeve-valve-pipe grouting technique has been extensively used in urban subway station construction projects in China. By injecting cement grout into the sandy strata, soil particles and grout are bonded together, and the void space within the soil particles are filled with grout [24].

**Figure 13:**
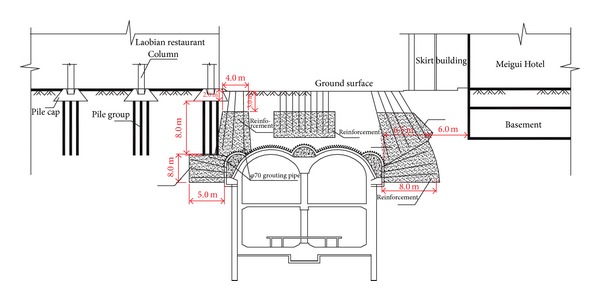
Sleeve-valve-pipe grouting technique.

The sleeve-valve-pipe grouting technique was adopted in the Zhongjie station project prior to the excavation to minimize ground settlement. The grouting was injected into the region between 3.0 m below the ground surface and 1.5 m above the tunnel crown and the thickness varies from 3.0 m to 5.0 m (see Figure 13) for the region between the subway station and existing surface buildings. Six-meter-long *φ*70 mm grout pipes were drilled from the ground surface toward the tunnel crown. The grout injection was conducted at a rate of 50 L/min. To prevent ground uplifting, the grouting pressure was strictly controlled within a range from 0.4 Mpa to 0.6 Mpa during the construction.

The related parameters of grouting included grout formula W∶C = 0.8∶1, incorporation of some accelerator, gel time of approximately 40 s, and a diffusion radius of approximately 0.7 m.

**Figure 14:**
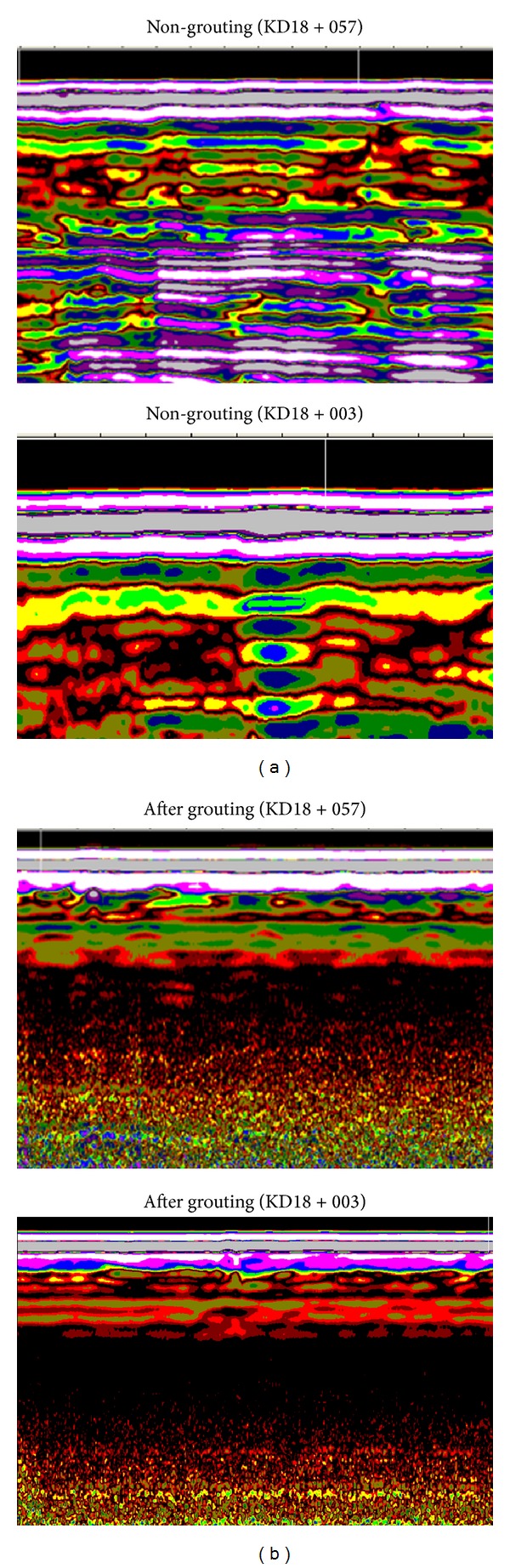
The comparison between grouting and non-grouting strata.

During construction, there were more than 1700 grout holes placed around the tunnel, with a minimum theoretical volume of 4500 m^3^. To determine the grouting quality, ground penetrating radar was applied in this project. Two cross sections of the detected ground compactness are shown in Figure 14. We can observe from the monitoring graph that the soil compactness increased significantly compared with the initial conditions. This indicates that the elastic modulus and strength of the strata increased effectively and improved self-bearing ability, which is helpful for restricting ground movements.

### 5.4. Reinforcement of the Building Foundation

**Figure 15:**
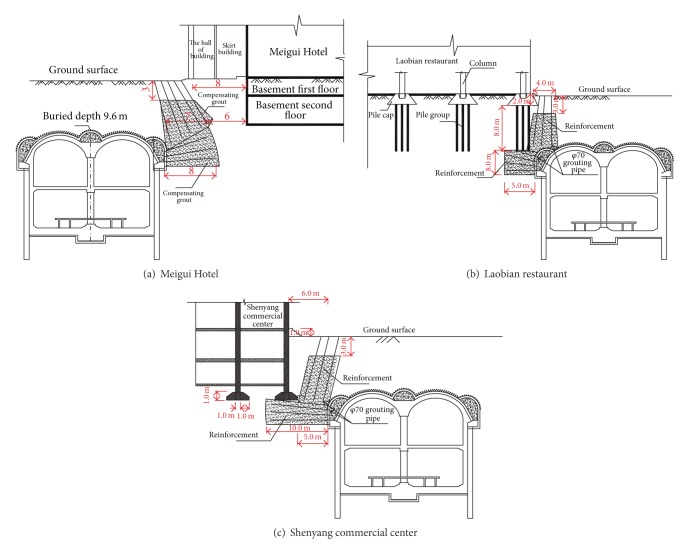
Reinforcement of the building foundation.

The excavation of subway stations will induce the movement of the foundation soil beneath the surface building toward the tunnel, which leads to the differential settlements of buildings or even collapse. The interaction between tunnel excavation and existing structure not only poses a potential risk to the underground project but also threatens the safety of surface buildings. To ensure the safety of tunnel and surface buildings, *φ*70 mm grout pipes were drilled from the inside of pilot headings toward the building foundation. The grouting pressure was controlled within 0.8 MPa to 1.0 MPa. Detailed foundation of building reinforcement schemes are illustrated in Figure 15.

### 5.5. Double-Layer Grouting Advanced Support

**Figure 16:**
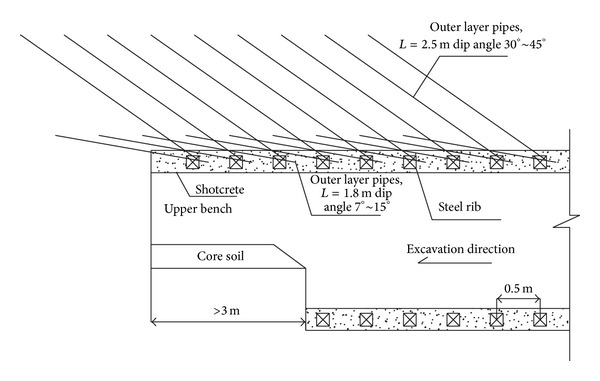
Double-layer grouting reinforcement.

The numerical simulation performed in Section 3.2 indicates that supporting arch construction is one of the key steps in the PBA excavation method. Double-layer forepoling pipes were implemented to strengthen the soil above the supporting arch prior to the excavation (see Figure 16) to ensure the safety of the tunnel and surface buildings during the supporting arch construction. The lengths of the inner layer pipe and outer layer pipe are 1.8 m and 2.5 m, respectively. The spacing in longitudinal and circumferential directions is 0.5 m and 0.3 m, respectively. The outer layer grouting pipes were injected with cement grout, whereas the inner layer grouting pipes were injected with sand solidification agent. Grouting pressure was controlled strictly within a range from 0.8 Mpa to 1.0 MPa in case of ground uplifting.

The main purpose of this type of double-layer grouting activity was to improve, stabilize, and strengthen the strata prior to the excavation. The mechanical parameters of the region between the tunnel crown and ground surface were improved by injecting cement grouts, and an umbrella above the tunnel crown was produced. This was helpful for restricting the ground movement and minimizing the adverse effects on the surrounding environments.

### 5.6. Backfill Grouting behind Primary Lining

During construction, there are plenty voids appearing behind the primary lining, which were created inadvertently. These voids are located in the region between linings and surrounding ground. To prevent the ground movements induced by the voids, the *φ*42 steel pipe, length of 0.6 m, was adopted to inject cement grout from inside of the pilot heading. Grouting pipes were arranged along the tunnel crown, and 2 m in longitudinal spacing.

## 6. Environmental Response to Tunneling

**Figure 17:**
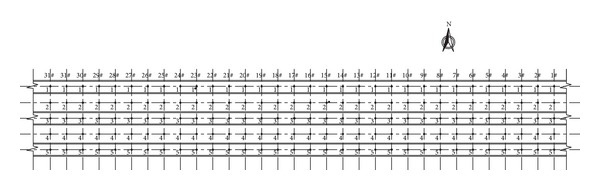
Plane view of the ground settlements monitoring points on a standard section.

Along the tunnel alignment, there are a number of surface buildings located around the Zhongjie subway station. During the construction stage, the safety of these buildings was one of the key problems encountered in this project. The monitoring scheme was designed to track building settlements and ground subsidence prior to the excavation (see Figures 9 and 17) to guarantee environmental safety.

The monitoring management standard of this project was classified into three grades: early warning value, alarm value, and limit value. Warning value is 70% that of the management critical value (shown in Table 2), whereas the alarm value and limit value are 85% and 100% that of the management critical value, respectively. The supporting patterns should be enhanced when the measured value reaches the early warning level. The excavation activities should be terminated immediately if the measured value reaches the limit level, and the evaluation of the safety situation of the surrounding buildings should be initiated.

### 6.1. Building Settlements

In this project, the settlement control indexes of the monitoring measurement value determined by the expert panel are shown in Table 3. The maximum settlement value for buildings was 40 mm. The settlement difference was controlled within 0.002 L (the length of existing building in the direction that is perpendicular to the tunnel axis). The control standard for the maximum ground surface settlement is 140 mm. Many shallow-buried projects in China have proven that the ground settlement allowable value should be controlled within 30 mm, which is unreasonable for a shallow-buried subway station.

**Figure 18:**
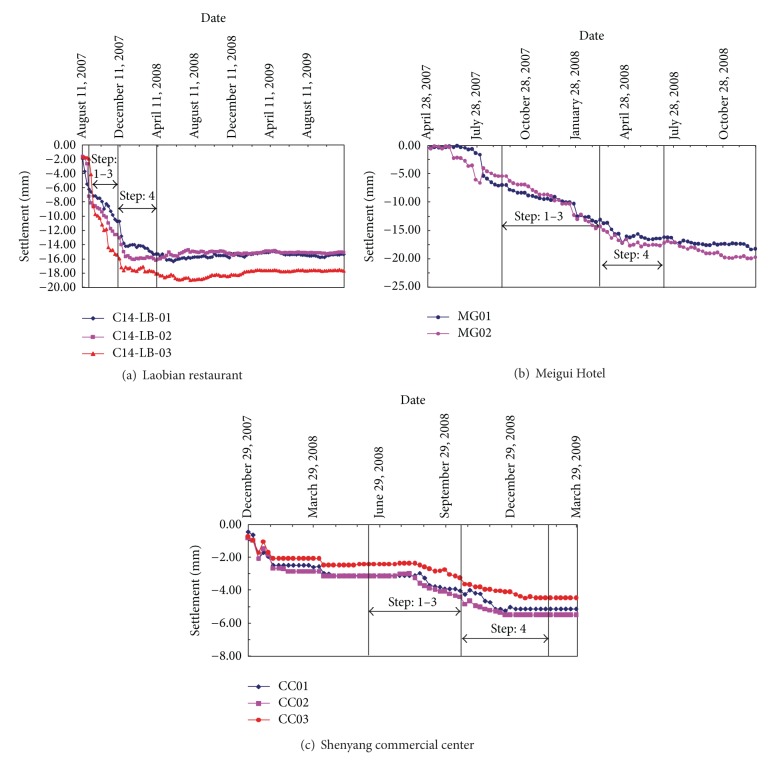
Long-term building settlement.

Settlement monitoring points were installed around each surface building. The settlements of these buildings were observed before, during, and after construction of the Zhongjie station. As shown in Figure 9, these buildings are located within the range of tunneling influence region. The settlements of the three buildings are shown in Figure 18. The maximum settlement of surface buildings induced by the excavation (including dewatering) was approximately 19.46 mm, which was observed with the Meigui Hotel. This is most likely because the Meigui Hotel is the highest building (20 stories, 70 m in height) among the affected surface buildings, and its foundation depth is relatively shallow (−7.5 m). Sirivachiraporn investigated the tunneling effects on buildings founded on different size pile lengths, and he stated that buildings on deep buried foundation displayed the least induced settlement [25]. In this project, the surface building that was least affected by the subway station excavation was the Shenyang commercial center, with a settlement of approximately 9.84 mm. This is mainly due to the structure having the deepest buried foundation depth (−11.6 m) among the affected buildings (see Table 2). The measured settlements of these buildings were controlled below 20 mm, and the deflection of these buildings was also smaller than 2 mm/m. This indicates that the risk mitigation measures had a significant effect on ground movement restriction. A further inspection of Figure 18 reveals that buildings can experience approximately 60% of their total settlement during the pilot heading excavation (from step 1 to step 3). This trend is similar to the numerical simulation presented in Section 3.2.

### 6.2. Ground Surface Settlements

During construction, there are approximately 320 (some of them were destroyed during the construction) monitoring points installed on the ground surface along the tunnel longitudinal direction. The monitoring points of a standard section are shown in Figure 17.

**Figure 19:**
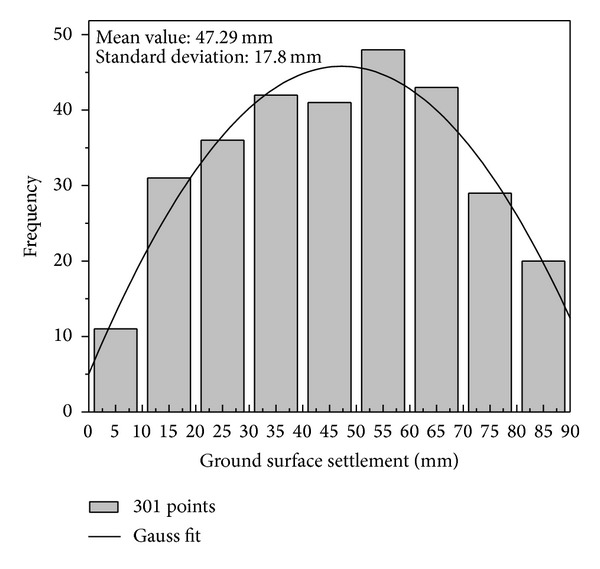
Statistical data of ground settlement induced by the subway station excavation.

The statistical data for ground surface subsidence caused by the subway station excavation in this project are shown in Figure 19. A further inspection of Figure 19 indicates that settlements generally exceeded 30 mm in 74.08% of the measurements. In addition, 6.64% of the measurements exceeded 80 mm. The maximum settlement was approximately 91.7 mm. The ground surface settlement for the measurements was successfully controlled below the early warning value, that is, 75% of that of the management critical value, which is equal to 105 mm.

**Figure 20:**
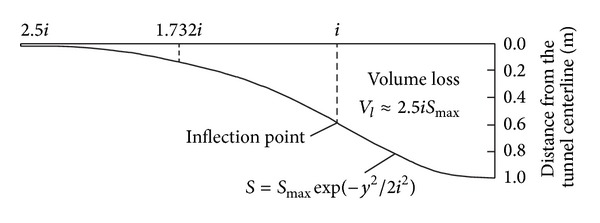
Transverse surface settlement trough curve.

Peck [26] reported that the transverse ground settlement profile can be expressed by a Gaussian distribution curve (see Figure 20). He derived the following empirical equation from their collected field measurement data:

(2) $$\begin{array}{c} S=S_{\max}\exp\left(\frac{-y^{2}}{\left(2i^{2}\right)}\right), \end{array}$$

where *S*_max⁡_ is the maximum ground surface settlement on the tunnel centerline, *y* is the horizontal distance from the tunnel centerline, and *i* is the horizontal distance from the tunnel centerline to the point of inflection on the settlement trough, which determines the shape and scope of the settlement trough.

The volume loss *V*_*l*_ induced by tunneling can be obtained by integrating (2) along the distance *y*, resulting in the following:

(3) $$\begin{array}{c} V_{l}=\overset{+\infty }{\underset{-\infty }{\int }}Sdy=\sqrt{2\pi }iS_{\max}\approx 2.5iS_{\max}. \end{array}$$

O'Reilly and New [27] proposed a relationship between tunnel buried depth *z*_0_ and *i*, based on the monitoring data obtained from a UK tunneling project. The linear function can be expressed as follows:

(4) $$\begin{array}{c} i=Kz_{0}, \end{array}$$

where *z*_0_ is the tunnel buried depth and *K* is the parameter of settlement trough, and its value is determined by stratigraphic condition.

**Figure 21:**
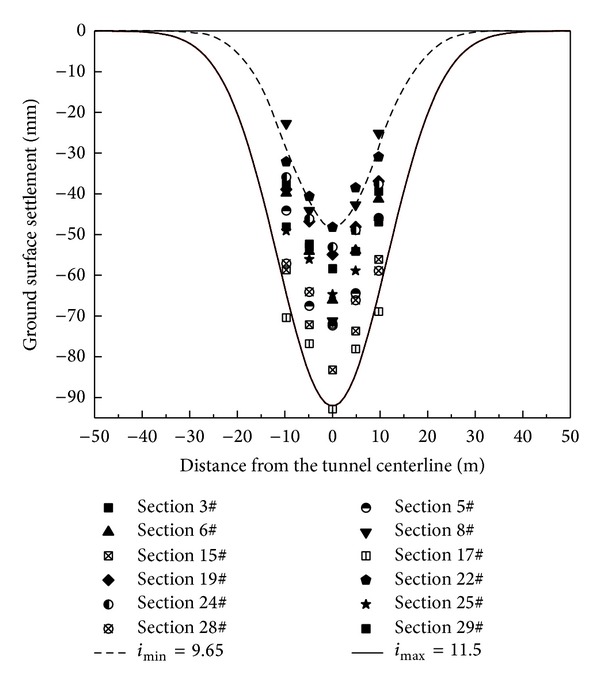
Fitting results of ground surface settlement trough.

The field observation data collected from 12 monitoring sections were fitted by a Gaussian distribution curve and are shown in Figure 21. It can be seen that the measured settlements are distributed in the area enclosed by the Gaussian fitting curves for *i* values ranging from 9.65 m (upper bound) to 11.5 m (lower bound), corresponding to 0.66–0.82 of transverse settlement trough parameter *K*. The tunnel volume losses *V*_*l*_ derived from (3) are in the range from 0.36% to 0.85%. This value is relatively small compared to the findings of other research reported by Mair [28] for open face tunneling in stiff clays, which were usually between 1% and 2%. This difference is mainly due to the different ground conditions and tunnel excavation methods.

## 7. Conclusions

The paper focused on the key techniques and risks management in a case study of the Zhongjie subway station, which was constructed in sandy soil using the PBA excavation method in a densely populated urban area. The main conclusions derived from the study are as follows.

1. The numerical simulation results indicate that. for a subway station constructed using the PBA excavation method, the excavation of pilot headings and the supporting arch constitute a high proportion of the ground surface settlement, 56.6% and 28.3% of the total settlement, respectively. Therefore, some risk mitigation measures should be undertaken during these excavation stages to restrict the ground movements and adverse impact on the surrounding environment.
2. Some risk mitigation measures were adopted in this project to ensure the environmental safety during the excavation, for example, appropriate groundwater dewatering scheme, feet-lock bolt, sleeve-pipe grouting technique, double-layer grouting pipe, and compensating grouting measures. These risk mitigation measures played a good role in ground movement control as no accidents occurred during or after the construction of the Zhongjie station.
3. After the construction was completed, the maximum deflection for the surface buildings was restricted below 2 mm/m, and the maximum surface building settlement was less than 20 mm. The surface building that was least affected by the subway station excavation was the Shenyang commercial center, which has a deep-buried foundation. The settlements of buildings on relatively shallow-buried foundation were larger than those of buildings on deep-buried foundation. The maximum ground settlement was 91.7 mm, which was also controlled below the early warning level, that is, equal to 105 mm.
4. Field observation data collected from 12 monitoring sections indicate that the transverse surface settlement measurements could subsequently be well fitted by a Gaussian distribution curve, and the settlement trough parameter *K* was in the range of 0.66 to 0.82, which corresponds to 0.36%~0.85% of tunnel volume loss. The relatively low volume loss indicates the effectiveness of several types of grouting measures on ground movement restriction.
5. Monitoring during shallow-buried subway station construction plays a significant role in environmental risks management. It is helpful to implement construction risk assessments at each stage of construction and provide available information for the decisions that need to be taken or modified at each construction step.

## Figures and Tables

**Table 1:** Mechanical parameter of soil layer and supporting structure.

Name	*h* (m)	*E* (Mpa)	*c* (kPa)	φ (°)	γ (kN/m)	*μ*
Miscellaneous fill	6.20	7.94	5	13	20.1	0.45
Silt sand	8.8	19.8	8.8	30.7	17.36	0.28
Medium sand	6.80	27.4	21.5	30.7	18.15	0.26
Coarse sand	4.4	27.4	21.5	34.7	18.15	0.26
Gravel sand	5.9	98	1.7	36.7	20.0	0.23
Primary lining	0.3	3000	/	/	25	0.25

γ: total unit weight; *E*: Young's modulus, which is obtained from the in situ tests; *h*: thickness of the soil layer; *μ*: Poisson's ratio of the soil.

**Table 2:** Detailed conditions of surface buildings.

Buildings	Foundation types/buried depth	Number of stories	Height/m	Construction time
Guanglu Cinema	Box foundation (−6 m)	6	20	1999
Women's world building	Box foundation (−7.5 m)	4	20	1995
Meigui Hotel	Box foundation (−7.5 m)	20	70	1987
Laobian restaurant	Pile foundation (−8 m)	7	25	1992
North Trade Building	Precast pile foundation (−8.5 m)	6	30	1994
Shenyang commercial center	Strip foundation (−11.6 m)	6	30	1991

**Table 3:** Management critical value for settlement of ground surface and building.

Construction step	Surface settlement	Building settlements		Horizontal convergence	Crown settlement
		Absolute	Differential		
Pilot heading excavation	80	25	—	10	50
Supporting arch	35	10	—	—	20
Main structure	25	5	—	15	—
Summation (mm)	140	40	0.002*L*	15	70

*L* denotes the length of the existing building in the direction perpendicular to the tunnel axis.

## References

[1] Chakeri H, Ozcelik Y, Unver B (2013). Effects of important factors on surface settlement prediction for metro tunnel excavated by EPB. *Tunnelling and Underground Space Technology*.

[2] Luo Y-B, Chen J-X (2013). Mechanical characteristics and mechanical calculation model of tunnel feet-lock bolt in weak surrounding rock. *Chinese Journal of Geotechnical Engineering*.

[3] Huang H (2006). State-of-the-art of the research on risk management in construction of tunnel and underground works. *Chinese Journal of Underground Space and Engineering*.

[4] Jurado A, de Gaspari F, Vilarrasa V (2012). Probabilistic analysis of groundwater-related risks at subsurface excavation sites. *Engineering Geology*.

[5] Eskesen SD, Tengborg P, Kampmann J, Veicherts TH (2004). Guidelines for tunnelling risk management: International Tunnelling Association, Working Group no. 2. *Tunnelling and Underground Space Technology*.

[6] Einstein HH (1996). Risk and risk analysis in rock engineering. *Tunnelling and Underground Space Technology*.

[7] Reilly JJ (2000). The management process for complex underground and tunneling projects. *Tunnelling and Underground Space Technology*.

[8] You K, Park Y, Lee JS (2005). Risk analysis for determination of a tunnel support pattern. *Tunnelling and Underground Space Technology*.

[9] Fang Q, Zhang D, Hou Y, Li B, Sun F (2010). Safety risk control technology of urban subway with shallow tunnel construction method. *Journal of Beijing Jiaotong University*.

[10] Wang T, Luo F-R, Liu W-N, Li X-G (2011). Influence of metro station construction by drift-pile-beam-arch method on soil and rigid-joint pipeline. *Rock and Soil Mechanics*.

[11] Wang T, Liu W, Zhang C, He H, Li X (2007). Study on ground settlement induced by shallow metro station constructions. *Chinese Journal of Rock Mechanics and Engineering*.

[12] Wang T, Luo F, Liu W, Li X (2012). Study of surface settlement and flexible joint pipeline deformation induced by metro station construction with PBA method. *China Civil Engineering Journal*.

[13] Yang Y, Weining L, Deyun D (2011). Analysis on heading excavation optimization in metro station constructed by drift-PBA method. *Chinese Journal of Underground Space and Engineering*.

[14] He H-J (2007 (Chinese)). *Influence and control of metro tunnelling by drift-PBA method on adjacent bridge piles [Ph.D. thesis]*.

[15] Fang Q, Zhang D, Wong LNY (2012). Shallow tunnelling method (STM) for subway station construction in soft ground. *Tunnelling and Underground Space Technology*.

[16] Liu J-F, Qi T-Y, Wu Z-R (2012). Analysis of ground movement due to metro station driven with enlarging shield tunnels under building and its parameter sensitivity analysis. *Chinese Journal of Underground Space and Engineering*.

[17] Shin JH, Lee IK, Lee YH, Shin HS (2006). Lessons from serial tunnel collapses during construction of the Seoul subway line 5. *Tunnelling and Underground Space Technology*.

[18] Forth RA (2004). Groundwater and geotechnical aspects of deep excavations in Hong Kong. *Engineering Geology*.

[19] Wang M-S (2006). Outline of tunnel construction by means of method of undercutting with shallow overburden. *Tunnel Construction*.

[20] Fraldi M, Guarracino F (2009). Limit analysis of collapse mechanisms in cavities and tunnels according to the Hoek-Brown failure criterion. *International Journal of Rock Mechanics and Mining Sciences*.

[21] Lei M, Peng L, Shi C (2014). Calculation of the surrounding rock pressure on a shallow buried tunnel using linear and nonlinear failure criteria. *Automation in Construction*.

[22] Wang M-S (1989). The boring excavation method and construetion in Beijing metro. *Chinese Journal of Rock Mechanics and Engineering*.

[23] Xiang Y, He S, Zhang M, Cui Z, Ma S (2004). Constraint effect of pilot-drift and separation-pile structure on ground movements induced by shallow tunneling. *Chinese Journal of Rock Mechanics and Engineering*.

[24] Wu S-C, Jin A-B, Gao Y-T (2007). Studies of sleeve-valve-pipe grouting technique and its effect on soil reinforcement. *Rock and Soil Mechanics*.

[25] Sirivachiraporn A, Phienwej N (2012). Ground movements in EPB shield tunneling of Bangkok subway project and impacts on adjacent buildings. *Tunnelling and Underground Space Technology*.

[26] Peck RB Deep excavation in soft ground.

[27] O’Reilly MP, New BM (1982). Settlements above tunnels in the United Kingdom—their magnitude and prediction. *Tunnelling*.

[28] Mair RJ Settlement effects of bored tunnels.

